# Chromosome-level genome assembly and annotation of the cold-water species *Ophiura sarsii*

**DOI:** 10.1038/s41597-024-03412-y

**Published:** 2024-05-30

**Authors:** Chen Han, Qian Zhang, Yixuan Li, Yuyao Sun, Yue Dong, Meiling Ge, Zhong Li, Xuying Hu, Bing Liu, Xuelei Zhang, Zongling Wang, Qinzeng Xu

**Affiliations:** 1 School of Ocean Sciences, China University of Geosciences, Beijing, 100083 China; 2 Key Laboratory of Marine Eco-Environmental Science and Technology, First Institute of Oceanography, Ministry of Natural Resources, Qingdao, 266061 China; 3 Faculty of Science, Hong Kong Baptist University, Hong Kong, 000000 China; 4 College of Environmental Science and Engineering, Ocean University of China, Qingdao, 266100 China

**Keywords:** Genome, Eukaryote

## Abstract

The cold-water species *Ophiura sarsii*, a brittle star, is a key echinoderm in the Arctic continental shelf region, highly sensitive to climate change. However, the absence of a high-quality genome has hindered a thorough understanding of its adaptive evolution. In this study, we reported the first chromosome-level genome assembly of *O. sarsii*. The genome assembly totalled 1.57 Gb, encompassing 19 chromosomes with a GC content of 37.11% and a scaffold N50 length of 78.03 Mb. The Benchmarking Universal Single-Copy Orthologs (BUSCO) assessment yielded a completeness estimate of 93.5% for this assembly. We predicted a total of 27,099 protein-coding genes, with 25,079 functionally annotated. The genome was comprised of 58.09% transposable elements. This chromosome-level genome of *O. sarsii* contributes to our understanding of the origin and evolution of marine organisms.

## Background & Summary

Global warming has led to increased ocean temperatures, resulting in significant transformations within marine ecosystems, particularly in the Arctic marginal seas^1^. *Ophiura sarsii*, belonging to the echinoderm phylum Ophiuroidea, is a predominant benthic organism in the Arctic continental shelf region, with a wide distribution across Japan, the Chukchi Sea, Canada, the United States, the North Atlantic, and Norway^1–3^. *Ophiura sarsii* constitutes crucial components of marine ecosystems, serving as key contributors to the marine biogeochemical cycle and providing essential support for a diverse array of ecosystem services^4–6^. This species plays integral roles in the food chain, exerting significant influence on energy flow and matter cycling within marine environments^7,8^. As a cold-water species, *O. sarsii* is sensitive to temperature fluctuations^9^, making it an ideal indicator for ecological assessments and a valuable model for studying species differentiation and evolution amid global environmental shifts^10^.

Meanwhile, *Ophiura sarsii* contains a diverse array of bioactive substances with medicinal potential, including chlorin compounds, which have demonstrated notable efficacy in photodynamic therapy against triple-negative breast cancer cells^11^. This discovery emphasizes the significance of *O. sarsii* in biomedicine as a source for novel cancer treatment compounds^11,12^. *Ophiura sarsii* exhibits robust regenerative capabilities, offering a critical model for scientific inquiry^13^. A comprehensive understanding of how these organisms efficiently regenerate lost appendages, encompassing intricate structures and components of the nervous system, yields valuable insights into the genetic and molecular mechanisms of regeneration^14,15^. This knowledge holds promise for informing research on human tissue regeneration and wound healing, potentially catalyzing advancements in the treatment of injuries and diseases involving tissue damage^13,15^. Additionally, *O. sarsii* is a deuterostome invertebrate, representing a key evolutionary link between achordates and chordates^16^, thus holding significant evolutionary status^17^. Nonetheless, the adaptive evolution of *O. sarsii*, despite its ecological significance, remains underexplored.

In this research, we presented the first chromosome-level genome assembly for *O. sarsii* by leveraging a hybrid approach that incorporates Illumina short-read sequencing (105.11 Gb), Pacbio Single Molecule Real-Time sequencing (40.08 Gb), and high-throughput chromatin conformation capture (Hi-C) sequencing (172.08 Gb) (Table 1). The resultant genome size is 1.57 Gb (Table 2), organized into 19 chromosomes (Table 3) with an N50 size of 78.03 Mb. Our data, the first chromosomal level data in ophiuroidea, can not only provide basic data for revealing the adaptive evolution mechanism of cold-water representative species and elucidating the origin and evolution of marine organisms, but also provides a theoretical basis for understanding the long-term environmental adaptability of cold-water species in the context of global warming.

**Table 1:** Statistics of the genome sequencing data of *O.sarsii*.

Library	Reads number	Raw data (bp)	Clean data (bp)	Reads N50	Q20(%)	GC Content (%)	Coverage
Illumina	351,128,543	105,338,562,900	105,113,985,600	/	96.62	37.55	99.63
Pacbio	3,415,506	/	40,078,903,730	12,313	/	36.99	97.70
Hi-C	1,216,271,086	182,440,662,900	172,084,446,898	/	96.38	38.49	/

**Table 2:** Summary statistics for *O.sarrsi* genome assembly.

Contig level	Value
Assembly length (bp)	1,592,177,811
Longest contig (bp)	1,741,681
Number of contigs	8,048
GC (%)	37.11
Contig N50 (bp)	311,019
Contig N90 (bp)	93,669
**Chromosome level**	**Value**
Number of chromosomes	19
Assembly length (bp)	1,574,763,175
Chromosome length (bp)	1,529,800,452
Scafold N50 (bp)	78,030,574
Loading rate (%)	97.14

**Table 3:** The length of each chromosome in *O.sarsii* genome.

Chromosome ID	Length (bp)
chr1	159,577,898
chr2	99,260,978
chr3	89,779,121
chr4	83,571,003
chr5	83,127,339
chr6	79,407,082
chr7	79,378,501
chr8	78,785,024
chr9	78,030,574
chr10	75,966,906
chr11	74,267,434
chr12	73,386,173
chr13	72,566,743
chr14	69,982,176
chr15	69,549,530
chr16	68,667,413
chr17	66,776,710
chr18	64,630,112
chr19	63,089,735

## Methods

### Sample collection and sequencing

The samples of *O. sarsii* were collected using box corers during the tenth Chinese Arctic expedition from August to September 2019^18^. Tissues from the arm base were excised, rinsed in 1X phosphate-buffered saline (PBS), and immediately preserved in liquid nitrogen before being stored at −80 °C. High-quality DNA was extracted using CTAB^19^ method for long-read and short-read whole genome sequencing. Total RNA was isolated using a commercial Animal Tissue Total RNA Extraction Kit (Tiangen, Beijing, China) according to the provided protocol.

For Illumina sequencing, genomic DNA was fragmented into 300-500 bp pieces, and a paired-end genomic library was prepared following the manufacturer’s protocol. Then, the library was sequenced on an Illumina HiSeq X-Ten platform using a paired-end 150 bp layout. For PacBio sequencing, the genomic DNA was used to construct SMRT bell libraries following the manufacturer’s protocol. After that, the libraries were sequencing on the PacBio Sequel II platform utilizing SMRT technology.

For the Hi-C sequencing, fresh tissue was crosslinked using 4% formaldehyde solution and digested with four-cutter restriction enzyme (*Mbo* I)^20^. The ends of the restriction fragments were labeled with biotinylated nucleotides (biotin-14-dCTP), and then the ligated DNA was sheared to 350 bp fragments for Hi-C library construction. The resulting library was quantified with the qRT-PCR method and sequenced with the Illumina HiSeq. 2500 (PE125) platform.

For transcriptome sequencing, total RNA of fresh tissue from *O. sarsii* was extracted for cDNA library construction. The resulting library was constructed by NEBNext® Ultra™ RNA Library Prep Kit (NEB, USA) according to the manufacturer’s instructions and sequenced on the Illumina HiSeq X-Ten platform.

### Genome size and heterozygosity estimation

**Fig. 1:**
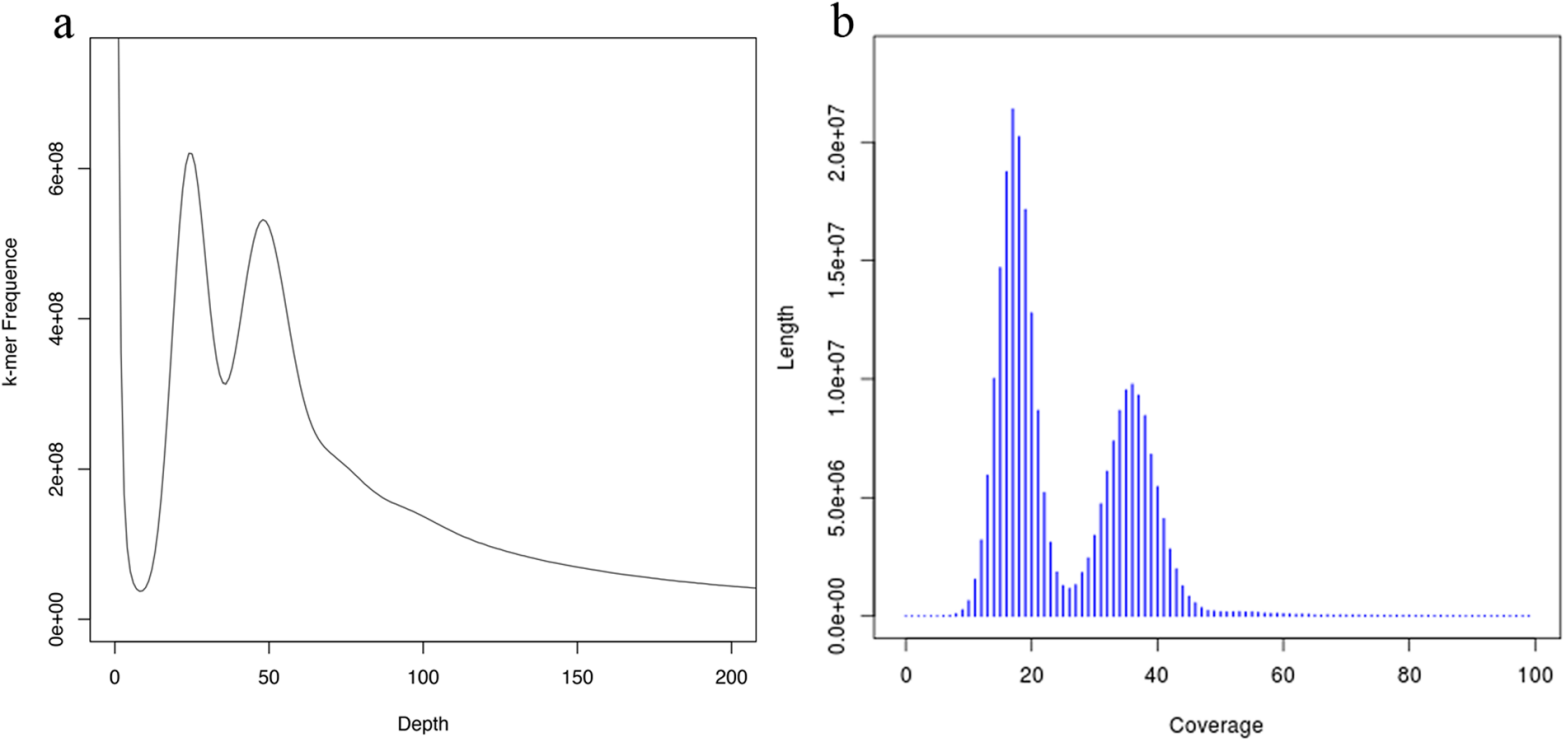
(**a**) The k-mer distribution used to estimate the genome size of *O. sarsii*. The distribution was determined based on the Jellyfish analysis using a k-mer size of 17. (**b**) Contig covers depth and length profiles of *O. sarsii*.

To ensure the quality of information analysis, strict filtering of Illumina sequencing data was performed using pk_qc.v2 and redup.v2 (proprietary software developed in-house by Novogene Co., LTD) with Default parameter, resulting in clean reads. The genome size of *O. sarsii* was estimated based on Illumina sequencing data using the k-mer counting method by the jellyfish 2.2.7^20^ with parameters of “-G 2 -m 17 -C”. Likewise, the heterozygosity rate was estimated utilizing the count of k-mers at half the peak depth. Based on the results of the survey analysis, the main peak was observed around depth  =  47 in Fig. 1(a). The genome size calculated using the formula Kmer-number/depth was approximately 1.59 Gb, and the adjusted genome size was 1.58 Gb. The genome heterozygosity rate was 1.97%, and the proportion of repetitive sequences was 63.40%. Utilizing Illumina data, an initial genome assembly of *O. sarsii* was conducted using the SOAPdenovo2 r242^21,22^ with parameters of “-K 41 -R -d 1”. Subsequently, the distribution of contigs was analyzed (Fig. 1(b)). The assembly was performed with a k-mer value of 41, yielding a contig N50 of 697 bp, resulting in a total length of 1.66 Gb. Furthermore, the scaffold N50 reached 975 bp, comprising a cumulative genome length of 1.73 Gb (Table 4).

**Table 4:** Assembly statistics for Illumina sequencing data.

Title	Total length (bp)	Total number	Max length (bp)	N50 length (bp)	N90 length (bp)
Contig	1,657,187,168	4,254,794	41,437	697	139
Scaffold	1,732,934,859	3,541,327	69,903	975	167

### *De novo* genome assembly

The PacBio HiFi reads of *O.sarsii* were *de novo* assembled by using Hifiasm v0.16.1^23^ with default parameters. A total of 40.08 Gb HiFi reads with N50 sizes of 12,313 bp were obtained using Circular Consensus Sequencing (CCS) mode (Table 1). The software Purge Haplotigs^24^ with default parameters was utilized for the purpose of de-redundancy in the genome after initial assembly correction. This involved the recognition and elimination of redundant heterozygous contigs based on both read depth distribution and sequence similarity. The draft genome had a total size of 1.59 Gb containing 8,048 contigs with N50 sizes of 311,019 bp (Table 2).

To confirm the assembly results belonging to the target species, the fragmented sequences were aligned to the NCBI Nucleotide Database (NT database) using Blast v2.4.0^25^ (Table 5). Sequencing data from Illumina was aligned to the reference genome using BWA v0.7.17^26^ with parameter of “bwa mem -k 19 -w 100”. Sequencing data from Pacbio was aligned to the reference genome using Minimap2 v2.24^27^ with parameter of “-x map-hifi”. Subsequently, the alignment rates and coverage were calculated for sequence consistency assessment (Table 6). In assessing the quality of the genome assembly, we appraised its integrity through the utilization of BUSCO v5.2.2^28^ with a comprehensive mammalian database (metazoa_odb10 database) (Table 7).

**Table 5:** Summary statistics of the fragmented sequences against the NCBI Nucleotide Database (NT database).

Genus	Blast number	Total (%)
*Asterias*	19,088	48.66
*Patiria*	2,848	7.26
*Strongylocentrotus*	2,004	5.11
*Eptatretus*	1,987	5.07
*Spilosoma*	1,039	2.65

**Table 6:** *De novo* genome assembly data consistency assessment.

Library	Mapping rate (%)	Coverage (%)	Coverage at least 4X (%)	Coverage at least 10X (%)	Coverage at least 20X (%)
Illumina	98.55	99.89	99.63	98.79	92.55
Pacbio	99.99	99.99	97.70	84.3	50.49

**Table 7:** BUSCO assessment statistics of *de novo* assembly.

Type	Assembly		Annotation	
	Proteins	Percentage (%)	Proteins	Percentage (%)
Complete BUSCOs	892	93.5	902	94.5
Complete Single-Copy BUSCOs	871	91.3	854	89.5
Complete Duplicated BUSCOs	21	2.2	48	5
Fragmented BUSCOs	11	1.2	15	1.6
Missing BUSCOs	51	5.3	37	3.9
Total BUSCO groups searched	954	100	954	100

### Chromosome assembly using Hi-C data

**Fig. 2:**
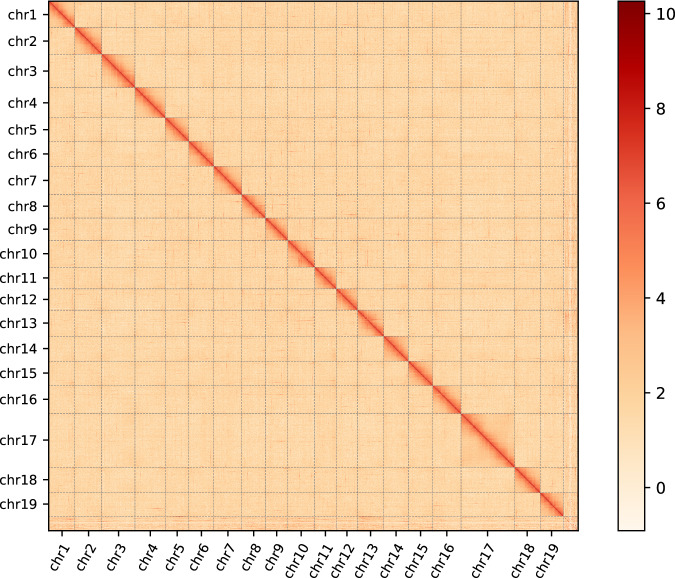
Genome-wide Hi-C heatmap of *O. sarsii*.

**Fig. 3:**
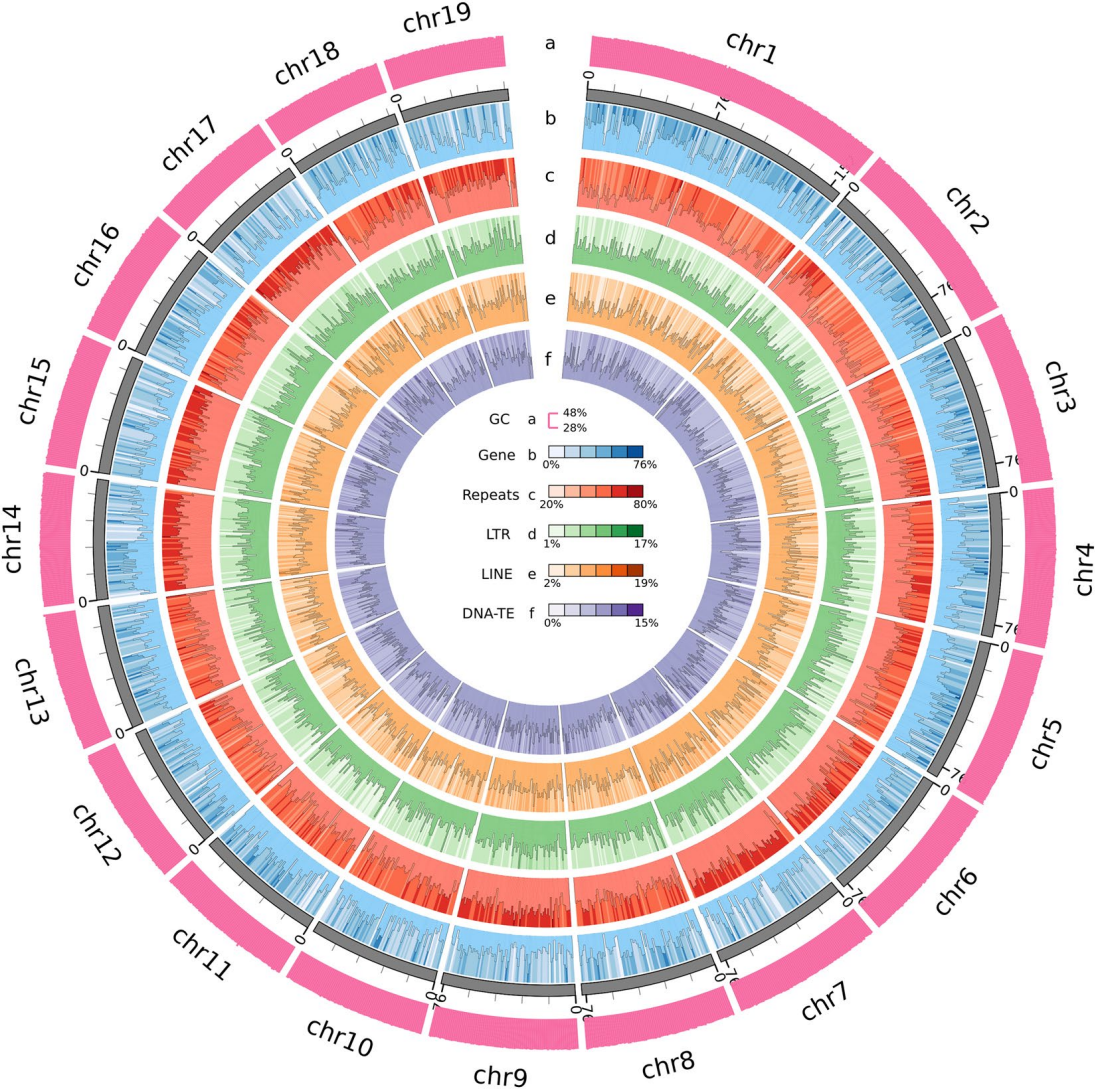
Characterization of the *O.sarsii* genome. From the outer to the inner layers, the GC density (**a**), gene density (**b**), repeat density (**c**), LTR density (**d**), LINE density (**e**) and DNA-TE density (**f**) are sequentially displayed.

The primary types of reads generated from Hi-C sequencing data encompass valid di-tags, contiguous sequences, circularized, dangling ends, internal fragments, PCR duplicates, and wrong sizes^29,30^. Hi-C sequencing data were subjected to filtration using HiCUP v0.8.0^31^ with default parameter. The clean Hi-C reads were then mapped to the contig assembly using Juicer v1.6^32^ with default parameter. Leveraging the principle that cis-interactions (interactions within the same chromosome) significantly surpass trans-interactions (interactions between distinct chromosomes), and considering the enhanced strength of cis-interactions with decreasing linear distance, the 3D-DNA pipeline^30^ with default settings is employed for the segmentation, anchoring, sorting, orientation, and amalgamation of contigs or scaffolds to obtain chromosomal-level genome. Following assisted genome assembly, the assembled genome underwent visualization and correction using JuiceBox v1.11.08^33^ to address potential errors in contig order, orientation, or internal assembly. As a result, Hi-C data facilitated the anchoring of contigs onto 19 chromosomes (Fig. 2). Circos v0.69-9^34^ was used to draw a circle diagram to describe the Characterization of the *O.sarsii* genome (Fig. 3).

### Genome annotation

Repetitive sequences in the *O. sarsii* genome were annotated through a synergistic approach, combining *de novo* and homology-based prediction methods. Tandem repeat sequences within the genomic DNA were discerned utilizing the TRF v4.09^35^. Transposable elements in the genomic sequence were annotated using RepeatMasker v4.1.2^36^, referencing the RepBase database v20181026. The resultant sequence file, generated through RepeatModeler v2.0^37^ (the ‘-LTRStruct’ option) and LTR-FINDER^38^, was employed as a library for the *de novo* prediction of repetitive elements in the genomic sequence using RepeatMasker v4.1.2^36^. A total of 914.83 Mb (58.09% of the genome) repetitive elements were identified (Table 8).

**Table 8:** Summary of repetitive sequences in the genome assembly of *O.sarsii*.

	Homolog		*De novo*		Combine	
	Length (bp)	Percent (%)	Length (bp)	Percent (%)	Length (bp)	Percent (%)
DNA	24,540,300	1.55	122,263,333	7.76	137,707,463	8.74
LINE	91,785,319	5.83	99,477,623	6.32	121,597,690	7.72
SINE	2,783,894	0.18	21,887,040	1.39	23,849,421	1.51
LTR	23,194,002	1.48	67,120,876	4.26	78,615,983	4.99
Satellite	1,349,777	0.09	10,235,876	0.65	11,456,044	0.73
Simple repeat	0	0	1,540,892	0.1	1,540,892	0.1
Other	10,309	0	0	0	10,309	0
Unknown	1,274,374	0.08	574,870,003	36.51	575,193,312	36.53
Total	142,424,456	9.05	870,065,385	55.25	914,825,359	58.09

The prediction of coding gene structures is conducted through an integrated approach, combining *de novo* prediction, homologous prediction and transcriptome-based prediction. *De novo* gene prediction was performed using Genscan^39^ and Augustus v3.4.0^40,41^ with default parameters. The pre-trained model of Augustus was pisaster. Homology-based prediction was performed using GEMOMA v1.9.0^42,43^ based on protein sequences of 7 echinoderm species (Table 9).

**Table 9:** The URLs for protein sequences of 7 species used for homology prediction.

Species	URL
*Strongylocentrotus purpuratus*	https://www.uniprot.org/proteomes/UP000007110
*Apostichopus japonicus*	https://www.ebi.ac.uk/ena/browser/view/GCA_002754855.1?show=blobtoolkit
*Asterias rubens*	https://ftp.ncbi.nlm.nih.gov/genomes/all/GCF/902/459/465/GCF_902459465.1_eAstRub1.3/
*Acanthaster planci*	https://ftp.ncbi.nlm.nih.gov/genomes/all/GCF/001/949/145/GCF_001949145.1_OKI-Apl_1.0/
*Anneissia japonica*	https://ftp.ncbi.nlm.nih.gov/genomes/all/GCF/011/630/105/GCF_011630105.1_ASM1163010v1/
*Ophiothrix exigua*	https://www.ncbi.nlm.nih.gov/bioproject/764446
*Lytechinus variegatus*	https://academic.oup.com/gbe/article/12/7/1080/5841217#206041273

For transcriptome-based prediction, RNA-seq reads were mapped to the genome using HISAT2 v2.2.1^44^ with default parameters and the transcriptome was assembled using STRINGTIE v2.2.0^45^. The open reading frames (ORFs) were predicted by TransDecoder v5.5.0^46^. ISO-seq reads were analyzed using StringTie2 v1.3.6^47^. Using default parameters of the MAKER v3.01.03, the results files of the above-mentioned software were added to the MAKER configuration file (maker_exe.ctl, maker_bopts.ctl, maker_opts.ctl, maker_evm.ctl) and the gene sets predicted by above methods were integrated into a non-redundant and more comprehensive gene set. Simultaneously, leveraging the integrated results using CEGMA v2.5^48^, the HiCESAP^49^ workflow was employed to derive the final reliable gene set. The final gene set annotation identified a total of 27,099 genes (Table 10). Finally, functional annotation of proteins within the gene set was accomplished through referencing external protein databases such as SwissProt (http://www.uniprot.org/), TrEMBL (http://www.uniprot.org/), KEGG (http://www.genome.jp/kegg/), InterPro (https://www.ebi.ac.uk/interpro/), and GO (http://geneontology.org/page/go-database) (Table 11). For the functional annotation of SwissProt and TrEMBL, BLAST v2.4.0 was employed for analysis with default parameters. KEGG, InterPro, and GO annotations were performed using KEGG API, InterProScan, and Blast2GO with default parameters, respectively.

**Table 10:** Summary of genome structure annotation in the genome assembly of *O.sarsii*.

Methods	Gene sete	Gene number	Average gene length(bp)	Average CDS length(bp)	Average exon per gene	Average exon length(bp)	Average intron length(bp)
*De novo*	Genscan	37,445	23,996	1,660	6.63	250.13	3,964
	AUGUSTUS	44,909	10,351	1,245	5.2	239.58	2,170
Homology	*Strongylocentrotus purpuratus*	26,582	15,704	1,128	5.09	221.44	3,562
	*Apostichopus japonicus*	34,079	8,584	833.58	3.4	245.29	3,232
	*Asterias rubens*	21,686	18,501	1,236	6.06	204.01	3,415
	*Acanthaster planci*	21,463	19,125	1,186	6.16	192.46	3,474
	*Anneissia japonica*	24,206	14,216	1,013	4.87	208.14	3,412
	*Ophiothrix exigua*	57,181	12,350	984.45	4.1	240.04	3,665
	*Lytechinus variegatus*	22,859	16,676	1,206	5.55	217.4	3,400
Transcript	RNAseq	6,992	25,854	1,438	7.26	435.68	3,625
	ISOseq	6,833	33,865	1,551	10.12	344.25	3,332
	MAKER	37,024	18,103	1,486	6.62	302.11	2,868
	HiCESAP	27,099	19,991	1,586	7.13	318.67	2,893

**Table 11:** Summary of genome function annotation in the genome assembly of *O.sarsii*.

Database	Gene number	Percent (%)
InterPro	21,842	80.6
GO	16,353	60.35
KEGG_ALL	22,877	84.42
KEGG_KO	12,820	47.31
Swissprot	16,691	61.59
TrEMBL	23,035	85
TF	1,703	6.28
Pfam	20,770	76.64
NR	23,805	87.84
KOG	15,903	58.68
Unannotated	2,020	7.45

Non-coding RNAs, such as tRNA, rRNA, miRNA and sn RNA were annotated. The tRNAscan-SE v2.0.5^50^ was employed to identify tRNA sequences in the genome based on the structural characteristics of tRNA. Due to the highly conserved nature of rRNA, BLASTN alignment is employed to search for rRNA in the genome. The prediction of miRNA and snRNA sequences on the genome is achieved using the covariance models from the Rfam family and INFERNAL provided by Rfam^51^ (Table 12). Based on the metazoa_odb10 database, BUSCO v5.2.2^28^ assessment was conducted on the annotated data (Table 7).

**Table 12:** Summary of ncRNA annotation in the genome assembly of *O.sarsii*.

Type		Copy	Average length(bp)	Total length(bp)	Percent (%)
miRNA		383	103	39,493	0.00251
tRNA		9,041	75	674,040	0.0428
rRNA	rRNA	473	129	61,114	0.00388
	18 S	4	1,235	4,941	0.00031
	5.8 S	2	157	314	0.00002
	5 S	467	120	55,859	0.00355
snRNA	snRNA	871	131	114,459	0.00727
	CD-box	294	104	30,674	0.00195
	HACA-box	29	147	4,270	0.00027
	splicing	548	145	79,515	0.00505

## Data Records

All raw sequencing data that were used for genome assembly and annotation have been deposited into the National Center for Biotechnology Information (NCBI) with accession number SRR27344560^52^ for Illumina sequencing data, SRR27353256^53^ and SRR27353257^54^ for Pacbio sequencing data, SRR27377125^55^, SRR27377126^56^ and SRR27377127^57^ for Hi-C sequencing data, SRR27371810^58^, SRR27371811^59^, SRR27371812^60^, SRR27371813^61^, SRR27371814^62^, SRR27371815^63^, SRR27371816^64^, SRR27371817^65^ and SRR27371818^66^ for RNAseq data, SRR27372082^67^ for ISOseq data. The genome assembly has been deposited at GenBank under the accession JAYJML000000000^68^. The version described in this paper is version JAYJML010000000.In addition, the final genome assembly data and annotation file is available in figshare^69^.

## Technical Validation

To validate the assembly results associated with the target species, the fragmented sequences were aligned against the NCBI Nucleotide Database (NT database) using Blast v2.4.025 (Table 5). The completeness of *O.sarsii* genome assembly was evaluated using the BUSCO (in the metazoa_odb10 database), and the completeness was 93.1% (86.2% single-copied genes and 6.8% duplicated genes), 1.6% fragmented, and 5.3% missing genes (Table 7). The Hi-C heatmap revealed a well-structured interaction pattern in and around the chromosome inversion regions (Fig. 3). All available evidence robustly supports the completeness and accuracy of *O.sarsii* genome assembly.

## Data Availability

All commands and pipelines employed in data processing were executed in strict accordance with the manuals and protocols of the respective bioinformatics software. In cases where detailed parameters were not explicitly provided, default parameters were applied. The version of each software utilized has been explicitly specified in the Methods section. No custom programming or coding was employed in the analysis.

## References

[1] Piepenburg D (2005). Recent research on Arctic benthos: common notions need to be revised. Polar Biology.

[2] Wang J (2014). Community structure and spatial distribution of macrobenthos in the shelf area of the Bering Sea. Acta Oceanologica Sinica.

[3] Grebmeier JM (2015). Ecosystem characteristics and processes facilitating persistent macrobenthic biomass hotspots and associated benthivory in the Pacific Arctic. Progress in Oceanography.

[4] Johnston CA, Gruner DS (2018). Marine fauna sort at fine resolution in an ecotone of shifting wetland foundation species. Ecology.

[5] Lessin G, Bruggeman J, McNeill CL, Widdicombe S (2019). Time scales of benthic macrofaunal response to pelagic production differ between major feeding groups. Front. Mar. Sci..

[6] Dong D (2021). Report of epibenthic macrofauna found from Haima cold seeps and adjacent deep-sea habitats, South China Sea. Mar. Life. Sci. Technol..

[7] Beck MW, Hatch LK (2009). A review of research on the development of lake indices of biotic integrity. Environ. Rev..

[8] Pelletier MC, Gold AJ, Heltshe JF, Buffum HW (2010). A method to identify estuarine macroinvertebrate pollution indicator species in the Virginian Biogeographic Province. Ecol. Indic..

[9] Hu G, Zhang Q (2016). Seasonal variations in macrobenthic taxonomic diversity and the application of taxonomic distinctness indices in Bohai Bay, northern China. Ecol. Indic..

[10] Bernard G, Gammal J, Jarnstrom M, Norkko J, Norkko A (2019). Quantifying bioturbation across coastal seascapes: habitat characteristics modify effects of macrofauna communities. J. Sea Res..

[11] Klimenko A (2023). Shallow- and Deep-Water Ophiura Species Produce a Panel of Chlorin Compounds with Potent Photodynamic Anticancer Activities. Antioxidants.

[12] McMurray JJV (2012). ESC Guidelines for the diagnosis and treatment of acute and chronic heart failure 2012 The Task Force for the Diagnosis and Treatment of Acute and Chronic Heart Failure 2012 of the European Society of Cardiology. Developed in collaboration with the Heart Failure Association (HFA) of the ESC. European Heart Journal.

[13] Czarkwiani A, Taylor J, Oliveri P (2022). Neurogenesis during Brittle Star Arm Regeneration Is Characterised by a Conserved Set of Key Developmental. Genes. Biology-Basel.

[14] Skold M, Rosenberg R (1996). Arm regeneration frequency in eight species of Ophiuroidea (Echinodermata) from European sea areas. Journal of Sea Research.

[15] Wood HL, Spicer JI, Lowe DM, Widdicombe S (2010). Interaction of ocean acidification and temperature; the high cost of survival in the brittlestar *Ophiura ophiura*. Marine Biology.

[16] Deline B (2020). Evolution and development at the origin of a phylum. Current Biology.

[17] O’Hara TD, Hugall AF, Woolley SN, Bribiesca-Contreras G, Bax NJ (2019). Contrasting processes drive ophiuroid phylodiversity across shallow and deep seafloors. Nature.

[18] Shi W (2021). Study on Genetic Diversity of *Ophiura sarsii* Populations in Arctic Region. Advances in Marine Science.

[19] Belton JM (2012). Hi-C: A comprehensive technique to capture the conformation of genomes. Methods.

[20] Marcais G, Kingsford C (2011). A fast, lock-free approach for efficient parallel counting of occurrences of k-mers. Bioinformatics.

[21] Luo R (2012). SOAPdenovo2: an empirically improved memory-efficient short-read de novo assembler. Gigascience.

[22] Li R (2010). De novo assembly of human genomes with massively parallel short read sequencing. Genome Research.

[23] Cheng H, Concepcion GT, Feng X, Zhang H, Li H (2021). Haplotype-resolved de novo assembly using phased assembly graphs with hifiasm. Nature Methods.

[24] Roach MJ, Schmidt SA, Borneman AR (2018). Purge Haplotigs: allelic contig reassignment for third-gen diploid genome assemblies. BMC Bioinformatics.

[25] Altschul SF, Gish W, Miller W, Myers EW, Lipman DJ (1990). Basic local alignment search tool. J. Mol. Biol..

[26] Li H (2013). Aligning sequence reads, clone sequences and assembly contigs with BWA-MEM. Genomics.

[27] Li H (2018). Minimap2: pairwise alignment for nucleotide sequences. Bioinformatics.

[28] Manni M, Berkeley MR, Seppey M, Simão FA, Zdobnov EM (2021). BUSCO Update: Novel and Streamlined Workflows along with Broader and Deeper Phylogenetic Coverage for Scoring of Eukaryotic, Prokaryotic, and Viral Genomes. Mol. Biol. Evol..

[29] Lieberman-Aiden E (2009). Comprehensive mapping of long-range interactions reveals folding principles of the human genome. Science.

[30] Dudchenko O (2017). De novo assembly of the Aedes aegypti genome using Hi-C yields chromosome-length scaffolds. Science.

[31] Steven W (2015). HiCUP: pipeline for mapping and processing Hi-C data. F1000Research.

[32] Durand NC (2016). Juicer provides a one-click system for analyzing loop-resolution Hi-C experiments. Cell Syst..

[33] Durand NC (2016). Juicebox provides a visualization system for Hi-C contact maps with unlimited zoom. Cell Syst..

[34] Krzywinski M (2009). Circos: an information aesthetic for comp arative genomics. Genome research.

[35] Benson G (1999). Tandem repeats finder: a program to analyze DNA sequences. Nucleic Acids Res..

[36] Chen N (2004). Using RepeatMasker to identify repetitive elements in genomic sequences. Curr. Protoc. Bioinformatics.

[37] Flynn JM (2020). RepeatModeler2 for automated genomic discovery of transposable element families. Proc. Natl. Acad. Sci..

[38] Xu Z, Wang H (2007). LTR_FINDER: an efficient tool for the prediction of full-length LTR retrotransposons. Nucleic Acids Res..

[39] Burge C, Karlin S (1997). Prediction of complete gene structures in human genomic DNA. Journal of Molecular Biology.

[40] Stanke M, Diekhans M, Baertsch R, Haussler D (2008). Using native and syntenically mapped cDNA alignments to improve de novo gene finding. Bioinformatics.

[41] Bruna T, Hoff KJ, Lomsadze A, Stanke M, Borodovsky M (2020). BRAKER2: automatic eukaryotic genome annotation with GeneMark-EP plus and AUGUSTUS supported by a protein database. Nar Genom. Bioinform..

[42] Keilwagen J (2016). Using intron position conservation for homology-based gene prediction. Nucleic Acids Res..

[43] Keilwagen J, Hartung F, Paulini M, Twardziok SO, Grau J (2018). Combining RNA-seq data and homology-based gene prediction for plants, animals and fungi. BMC Bioinformatics.

[44] Kim D, Paggi JM, Park C, Bennett C, Salzberg SL (2019). Graph-based genome alignment and genotyping with HISAT2 and HISAT-genotype. Nat. Biotechnol..

[45] Pertea M, Kim D, Pertea GM, Leek JT, Salzberg SL (2016). Transcript-level expression analysis of RNA-seq experiments with HISAT, StringTie and Ballgown. Nat. Protoc..

[46] Haas BJ (2013). De novo transcript sequence reconstruction from RNA-seq using the Trinity platform for reference generation and analysis. Nat. Protoc..

[47] Pertea M (2015). StringTie enables improved reconstruction of a transcriptome from RNA-seq reads. Nat. Biotechnol..

[48] Parra G, Bradnam K, Korf I (2007). CEGMA: a pipeline to accurately annotate core genes in eukaryotic genornes. Bioinformatics.

[49] Sun WJ (2020). Biofilm-Related, Time-Series Transcriptome and GenomeSequencing in Xylanase-Producing *Aspergillus niger* SJ1. Acs Omega.

[50] Lowe TM, Eddy SR (1997). tRNAscan-SE: a program for improved detection of transfer RNA genes in genomic sequence. Nucleic Acids Research.

[51] Ambros V (2003). A uniform system for microRNA annotation. Rna.

[52] (2024). NCBI Sequence Read Archive.

[53] (2024). NCBI Sequence Read Archive.

[54] (2024). NCBI Sequence Read Archive.

[55] (2024). NCBI Sequence Read Archive.

[56] (2024). NCBI Sequence Read Archive.

[57] (2024). NCBI Sequence Read Archive.

[58] (2024). NCBI Sequence Read Archive.

[59] (2024). NCBI Sequence Read Archive.

[60] (2024). NCBI Sequence Read Archive.

[61] (2024). NCBI Sequence Read Archive.

[62] (2024). NCBI Sequence Read Archive.

[63] (2024). NCBI Sequence Read Archive.

[64] (2024). NCBI Sequence Read Archive.

[65] (2024). NCBI Sequence Read Archive.

[66] (2024). NCBI Sequence Read Archive.

[67] (2024). NCBI Sequence Read Archive.

[68] (2024). NCBI GenBank.

[69] Chen H (2024). Figshare.

